# Gene Panel Sequencing Identifies a Novel *RYR1* p.Ser2300Pro Variant as Candidate for Malignant Hyperthermia with Multi-Minicore Myopathy

**DOI:** 10.3390/genes13101726

**Published:** 2022-09-26

**Authors:** Young Jae Moon, Joonhong Park, Jung Ryul Kim, Seung Yeob Lee, Jaehyeon Lee, Yong Gon Cho, Dal Sik Kim

**Affiliations:** 1 Department of Orthopedic Surgery, Jeonbuk National University Medical School and Hospital, Jeonju 54907, Korea; 2 Department of Biochemistry and Molecular Biology, Jeonbuk National University Medical School, Jeonju 54907, Korea; 3 Department of Laboratory Medicine, Jeonbuk National University Medical School and Hospital, Jeonju 54907, Korea; 4 Research Institute of Clinical Medicine of Jeonbuk National University-Biomedical Research Institute of Jeonbuk National University Hospital, Jeonju 54907, Korea

**Keywords:** *RYR1* variant, c.6898T > C, p.Ser2300Pro, malignant hyperthermia, multi-minicore myopathy, torticollis, ryanodine receptor-mediated calcium release, gene panel sequencing

## Abstract

Malignant hyperthermia (MH), a rare autosomal dominant pharmacogenetic disorder of skeletal muscle calcium regulation, is triggered by sevoflurane in susceptible individuals. We report a Korean having MH with multi-minicore myopathy functionally supported by RYR1-mediated intracellular Ca^2+^ release testing in B lymphocytes. A 14-year-old boy was admitted for the evaluation of progressive torticollis accompanied by cervicothoracic scoliosis. During the preoperative drape of the patient for the release of the sternocleidomastoid muscle under general anesthesia, his wrist and ankle were observed to have severe flexion contracture. The body temperature was 37.1 °C. To treat MH, the patient was administered a bolus of dantrolene intravenously (1.5 mg/kg) and sodium bicarbonate. After a few minutes, muscle rigidity, tachycardia, and EtCO2 all resolved. Next-generation panel sequencing for hereditary myopathy identified a novel *RYR1* heterozygous missense variant (NM_000540.2: c.6898T > C; p.Ser2300Pro), which mapped to the MH2 domain of the protein, a hot spot for MH mutations. Ex vivo RYR1-mediated intracellular Ca^2+^ release testing in B lymphocytes showed hypersensitive Ca^2+^ responses to isoflurane and caffeine, resulting in an abnormal Ca^2+^ release only in the proband, not in his family members. Our findings expand the clinical and pathological spectra of information associated with MH with multi-minicore myopathy.

## 1. Introduction

Malignant hyperthermia (MH, OMIM #145600), a rare autosomal dominant pharmacogenetic disorder of skeletal muscle calcium regulation, is triggered by sevoflurane in susceptible individuals [1,2]. Triggering substances can cause the uncontrolled release of calcium from the sarcoplasmic reticulum. They can promote the entry of extracellular calcium into the myoplasm, causing the contracture of skeletal muscles, glycogenolysis, and increased cellular metabolism, resulting in the production of heat and excess lactate. During an episode of MH, clinical manifestations occur as a result of the unregulated accumulation of myoplasmic calcium, which can lead to sustained muscular contraction and breakdown (rhabdomyolysis), cellular hypermetabolism, anaerobic metabolism, acidosis, and their sequelae [3]. Without proper and prompt treatment with dantrolene sodium, mortality is extremely high [4,5]. The diagnosis of MH is established with in vitro muscle contracture testing by measuring the contracture responses of biopsied muscle samples to halothane and graded concentrations of caffeine or halothane [6]. The diagnosis of MH can also be established by identifying a pathogenic variant in *RYR1*, *CACNA1S*, or *STAC3* genes in molecular genetic testing [7]. *RYR1* gene encodes for a ryanodine receptor of the skeletal muscle Ca^2+^ release channel and a voltage-gated dihydropyridine receptor. *CACNA1S* gene encodes the voltage-gated calcium channel α-subunit Cav1.1 and has an important role in Ca^2+^-mediated excitation-contraction coupling [8]. Mutations in *RYR1* and *CACNA1S* genes account for 50 to 70% of MH cases [7]. However, according to the European Malignant Hyperthermia Group (https://emhg.org/genetics/, accessed on 16 January 2022), currently, only 48 reported *RYR1* mutations and two *CACNA1S* mutations have proven pathogenic according to those stringent criteria [9,10].

To date, 16 cases of MH with *RYR1* mutations in 13 unrelated Korean families have been reported; however, a functional assay for analysis of variants of uncertain significance in *RYR1* was not studied [11,12,13,14]. We report a Korean with MH having multi-minicore myopathy functionally supported by ex vivo RYR1-mediated intracellular Ca^2+^ release testing in B lymphocytes.

## 2. Materials and Methods

### 2.1. Muscle Biopsy

After washing and draping the proximal 1/3 of the anterolateral tibia, a local anesthetic (lidocaine 1%) was injected skin area taking care not to infiltrate into the muscle. About a 3 cm skin incision was made with a pointed scalpel blade, and the skin and subcutaneous fat tissue on both sides of the incision were retracted. The fascia of the tibialis anterior (TA) muscle was incised, and 1 cm × 0.5 cm of the TA muscle was biopsied using a Metzenbaum scissor. After irrigation, the skin was sutured appropriately, and a dressing was applied. A fresh TA muscle specimen was freeze-fixed using isopentane and liquid nitrogen within 30 min.

### 2.2. Succinate Dehydrogenase Staining

The tibialis anterior muscle was placed in a 30% sucrose solution and embedded in liquid nitrogen-cooled isopentane. Frozen sections (8-µm thick) were incubated in 0.2 M sodium phosphate-buffered solution (pH 7.6) containing 0.6 mM nitro blue tetrazolium and 50 mM sodium succinate (Sigma-Aldrich, St. Louis, MO, USA) for 20 min at 37 °C. The slides were washed with DiH2O and mounted with aqueous mounting media.

### 2.3. Gene Panel Sequencing

To determine the potential genetic cause of the suspected MH in our proband, his genomic DNA was analyzed by gene panel sequencing using a Celemics G-Mendeliome Hereditary Myopathy Panel (Celemics, Seoul, Korea) (Table S1 in Supplementary Materials). Paired end (PE) sequencing was conducted using a NextSeq500 instrument (Illumina, San Diego, CA, USA) with a high output flow cell and 300 PE cycles (150 × 2) at the Green Cross Genome (Yongin, Korea) to detect the variant given the suspicion of a hereditary myopathy. Base-calling, alignment, variant calling, annotation, and quality control reporting were performed using the Genome Analysis Tool Kit best-practice pipeline workflow for germline short variant discovery (https://gatk.broadinstitute.org/hc/en-us/, accessed on 13 May 2021). DNA sequencing reads were aligned to the human genome reference assembly GRCh38 (hg38) using Burrows-Wheeler Aligner (BWA). Gene panel sequencing generated a yield of 226,451,035 target reads in the proband’s sample by estimating the sequence quality along all sequences. The mean read depth (x) was 195. The percentage of bases above a read depth of 30x was 99.8%. The interpretation of sequence variants was manually reviewed by medical laboratory geneticists according to the standards and guidelines from the Joint Consensus Recommendation of the American College of Medical Genetics and Genomics (ACMG) and the Association for Molecular Pathology (AMP) for classifying pathogenic variants [15]. Particularly, the pathogenic effect of missense variants was estimated using in silico computational tools (Polyphen2, http://genetics.bwh.harvard.edu/pph2/, accessed on 8 February 2022; MutationTaster, https://www.mutationtaster.org/, accessed on 8 February 2022; Evola, http://www.h-invitational.jp/evola/, accessed on 16 September 2022), Rare Exome Variant Ensemble Learner (REVEL) [16]. The predicted conservation scores of rare variants were determined using PhyloP (https://ccg.epfl.ch/mga/mm9/phylop/phylop.html/, accessed on 16 September 2022), GERP (http://mendel.stanford.edu/SidowLab/downloads/gerp/index.html/, accessed on 16 September 2022), and SiPhy (https://portals.broadinstitute.org/genome_bio/siphy/index.html/, accessed on 16 September 2022), and tests. The allele frequencies of rare variants in the general population and Korean ethnic population were estimated by the genome aggregation database (gnomAD, https://gnomad.broadinstitute.org/, accessed on 8 February 2022) and Korean Reference Genome Database (KRGDB, http://152.99.75.168/KRGDB/, accessed on 8 February 2022).

### 2.4. Sanger Sequencing

The presence of the *RYR1* variant was confirmed with bidirectional Sanger sequencing using primer pair of 5′-aggtctcaagctcctgttca-3′ and 5′-tcgagggaggtgtgtgac-3′ on a 3730xl DNA Analyzer (Applied Biosystems, Foster City, CA, USA). Segregation analysis was also performed to determine carrier status for family members using Sanger sequencing.

### 2.5. Ex Vivo RYR1-Mediated Intracellular Ca^2+^ Release Testing in B Lymphocytes

A volume of 10 mL of fresh peripheral blood was drawn from the proband and his family members. Peripheral blood mononuclear cells (PBMCs) were isolated by Ficoll-Hypaque density gradient centrifugation. For infection with Epstein–Barr virus, PBMCs were exposed to supernatants of the B95.8 cell line in the presence of interleukin (IL)-6 and cyclosporin A, according to standard procedures. Cells were cultured in RPMI medium supplemented with 2 mM L-glutamine, 10% fetal bovine serum (FBS), and 100 units of streptomycin and penicillin. The EBV-immortalized B cells were observed after 2 to 3 weeks. Then, the cells were passaged in standard RPMI 1640 medium containing 20% fetal bovine serum without any supplement change every 3 to 4 days [17,18]. The measurement of RyR1-mediated intracellular Ca^2+^ ([Ca^2+]^i) release response to isoflurane and caffeine can differentiate between MH-susceptible individuals and normal controls [19]. To measure cytosolic [Ca^2+^]i, single floating B lymphocytes were attached to Matrigel matrix (BD Biosciences, Bedford, MA, USA)-coated dishes. The culture medium was replaced with Dulbecco’s Modified Eagle Medium (DMEM) containing the membrane-permeable Ca^2+^ indicator Fluo-4 AM (Invitrogen, Carlsbad, CA, USA). [Ca^2+^]i and measured with a confocal microscope (Nikon eclipse C1, JAPAN). Calculations were performed using an equation by Tsien et al. [20]: [Ca^2+^]i = Kd (F − Fmin)/(Fmax − F), where Kd was 335 nM for Fluo 4 and F was the observed fluorescence level. Each tracing was calibrated for maximal intensity (Fmax) by adding ionomycin (8 μM) and for minimal intensity (Fmin) by adding 50 mM EGTA at the end of each measurement. To measure sarcoplasmic reticulum (SR) Ca^2+^ release, SR Ca^2+^ release was measured by the method with modifications described previously [21]. The kinetics of Ca^2+^ release was monitored under the standard condition at 25 °C in a medium containing 20 mM MOPS-Tris (pH 6.8), 5 mM MgCl_2_, 5 mM sodium oxalate and 20 nM Flou-2. The Final concentration of SR vesicle was maintained at 200 Mg/mL for all experiments. Fluorescence was recorded in a 1 cm cuvette with continuous magnetic stirring, using a Photon Technology International (PTI) spectrofluorometer. Simultaneous recordings were obtained at 0.85 Hz, and data was collected and analyzed with the PTI computer interface.

## 3. Case Presentation

**Figure 1:**
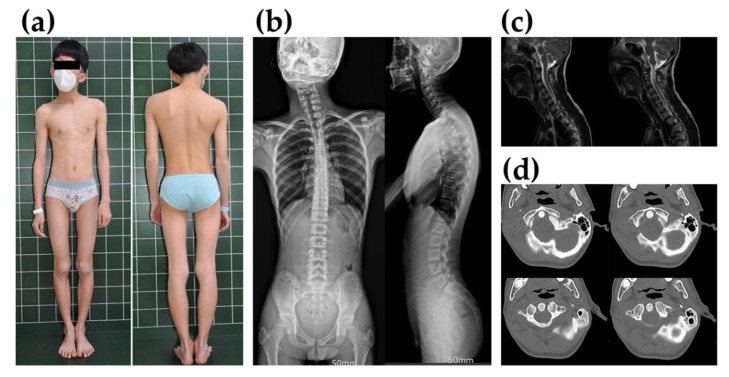
The proband’s phenotype and radiologic findings. (**a**) The proband with uncorrected muscular torticollis before surgery. His head is tilted toward the left, showing shoulder elevation and facial asymmetry. (**b**) Simple cervical, thoracic, and lumbar spine radiography. Cervicothoracic curvature was observed due to torticollis. (**c**) In T2-weighted sagittal magnetic resonance imaging (MRI) of the cervical spine, there were no abnormalities in the spinal cord and cervical disc. (**d**) Normal atlanto-axial (C1-C2) joint findings on computed tomography (CT).

A 14-year-old boy was admitted to Jeonbuk National University Hospital (Jeonju, Korea) for orthopedic surgery to evaluate progressive torticollis accompanied by cervicothoracic scoliosis. He was the first child of healthy, non-consanguineous Korean parents. There was no personal or family history of problems with anesthesia or intolerance to exercise or heat, neuromuscular disorders, or drug allergies. On physical examination, his head was tilted toward the left, with shoulder elevation, facial asymmetry, sternocleidomastoid muscle (SCM) tightness (Figure 1a,b), and uncorrected muscular torticollis. Although both elbow flexion, extension, and ankle dorsiflexion were observed in manual muscle testing grade 4+, there were no gait abnormalities or sensory problems. Computed tomography (CT) and magnetic resonance imaging (MRI) showed no abnormal findings on the spine and scapula (Figure 1c,d). The patient was considered to have neglected congenital muscular torticollis. We planned surgical intervention for the release of the SCM muscle under general anesthesia. The preoperative evaluation showed no abnormal findings. The patient was induced with thiopental sodium. General anesthesia was maintained with sevoflurane. During the preoperative drape of the patient, his wrist and ankle were observed to have severe flexion contracture. The EtCO2 increased to 74 mm Hg at that time. The heart rate was 160 beats/min. arterial blood gas (ABG) analysis showed lactic acidosis (lactate = 4.1, pH = 7.16) with hypercapnia (pCO2 = 73 mm Hg). The body temperature was 37.1 °C. Based on the patient’s symptoms, we suspected MH. Thus, we did not perform surgery and discontinued the sevoflurane. For the treatment of MH, the patient was administered a bolus of dantrolene intravenously (IV) (1.5 mg/kg) and sodium bicarbonate. After a few minutes, muscle rigidity, tachycardia, and EtCO2 all resolved. Laboratory tests showed increased creatine kinase (CK) (17,481 IU/L), lactate dehydrogenase (LD) (1020 IU/L), and aldolase (89.3 U/L) levels. Although generalized weakness and muscle soreness were observed for up to two days afterward, the patient made a full recovery and was discharged to his home. Based on the MH clinical grading scale [22], the proband scored 48 points: generalized muscle rigidity, 15 points; muscle breakdown with creatine kinase (CK) > 10,000 units/L, 15 points; respiratory acidosis with end-tidal CO_2_ > 55 mmHg and PaCO_2_ > 60 mmHg, 15 points; and cardiac involvement with sinus or ventricular tachycardia, 3 points. Thus, the MH rank of the proband was 5 with a “very likely” probability.

## 4. Results

**Figure 2:**
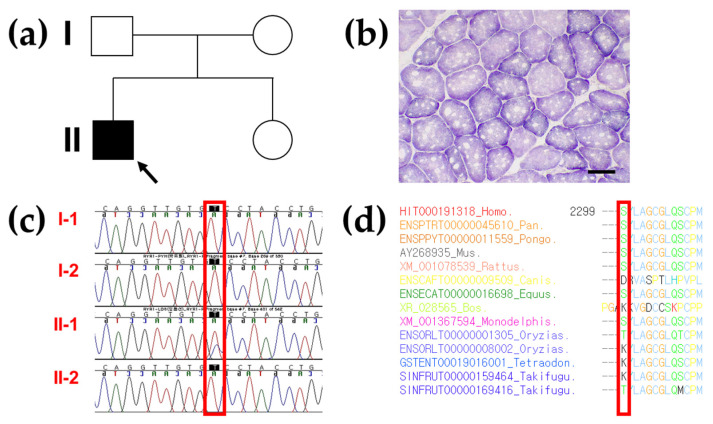
Pedigree analysis, succinate dehydrogenase staining, genetic analysis, and conservation study of the *RYR1* variant in the proband. (**a**) Pedigree of the proband with the heterozygous *RYR1* variant in an autosomal dominant manner (arrow) and his family members. (**b**) Succinic dehydrogenase staining (bar = 50 µm) of the tibialis anterior muscle biopsy in the proband demonstrating 5 or more eccentric multi-minicores in several fibers with various sizes and numbers, showing foci of decreased or absent enzymatic activity. (**c**) Sanger sequencing confirmed a heterozygous missense variant (NM_000540.2:c.6898T > C; p. Ser2300Pro) in *RYR1* occurring as de novo constitutional event in the proband (II-1) only. It is highlighted in a red box. (**d**) Sequence alignment of the conserved cytoplasmic domain of the RYR1 protein in multiple vertebrate species by Evola. The Ser2300 residue is highlighted in a red box.

To verify the association of MH with myopathy, electrodiagnostic testing was performed on the proband. Motor and sensory nerve conduction velocities were normal in the extremities. However, the electromyographic study showed abnormal spontaneous activity in all extremities, and the motor unit analysis study showed low amplitude and short duration in all extremities. To confirm MH-related myopathy, a muscle biopsy was performed under local anesthesia. Succinic dehydrogenase (SDH) staining of the muscle biopsy from the proband (II-1 in Figure 2a) demonstrated 5 or more eccentric multi-minicores in several fibers with various sizes and numbers, showing foci of decreased or absent enzymatic activity (Figure 2b).

After excluding variants with a population allele frequency >0.001 in the gnomAD, heterozygous missense variants of the three different genes were identified as a genetic cause of hereditary myopathy by gene panel sequencing in the proband. Among them, *RYR1* heterozygous missense variant, c.6898T > C/p.Ser2300Pro (reference transcript ID of *RYR1*: NM_000540.2) was the best candidate as the cause of autosomal dominant MH. Because the proband’s parents and his younger brother presented with no clinical symptoms associated with MH, genetic counseling and segregation analysis were estimated to identify the genetic cause of MH. As a result, Sanger sequencing confirmed the genetic origin of the *RYR1* variant as de novo (Figure 2c). In addition, the *GAA* and *GNE* heterozygous variants were excluded because clinical features and inheritance patterns did not match the patient (Table 1).

Paternity and kinship analysis was conducted using short tandem repeat (STR) multiplex assay (AmpFLSTR Identifiler; Applied Biosystems), and STR analysis confirmed the biological association between the proband and his parents. Multiple lines of computational evidence support the deleterious effect of this rare *RYR1* variant: It was predicted to be disease-causing by MutationTester and REVEL (score of 0.725), and possibly damaged according to PolyPhen-2 (score of 0.586). In addition, the protein sequence of the Ser2300 residue was conserved between humans and Takifugu except for the Canis, Bos, Monodelphis, Oryzias, Tetraodon, and Takifugu genera (Figure 2d). The predicted conservation scores of this rare variant were phyloP100 with a value of 0.67, SiPhy 29 way with a value of 8.435, and GERP with a value of 4.11 (Deleterious cutoff, PhyloP > 1.6; SiPhy > 12.17; GERP > 2). The allele frequencies of this rare variant in the general population and Korean ethnic population were observed. Thus, Novel *RYR1* p.Ser2300Pro Variant was presumptively classified as PM2 (Absent from controls in Exome Sequencing Project, 1000 Genomes or ExAC) by original ACMG-AMP criteria, however, not PP3 (Multiple lines of computational evidence support a deleterious effect on the gene or gene product) because of conflicting results of in silico computational analysis.

**Figure 3:**
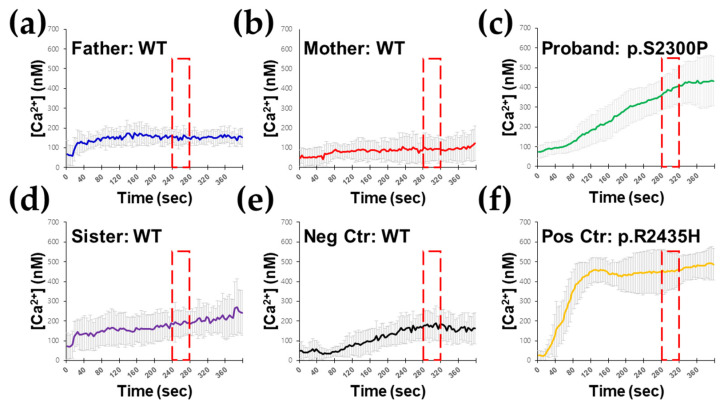
Ex vivo RYR1-mediated intracellular Ca^2+^ release testing response to isoflurane in B lymphocytes. B lymphocytes isolated from the wild-type (WT) proband’s father (**a**), WT proband’s mother (**b**), and WT proband’s sister (**d**) did not show hypersensitive Ca^2+^ responses to isoflurane. However, B lymphocytes isolated from the proband (**c**) demonstrated hypersensitive Ca^2+^ responses to isoflurane, resulting in an abnormal Ca^2+^ release. (**e**) Unaffected healthy individual as negative control (Neg Ctr). (**f**) B lymphocytes with p.Arg2435His mutation of the *RYR1* gene as positive control (Pos Ctr).

To prove the functional effect of this variant, RYR1-mediated [Ca^2+^]I release testing in B lymphocytes was performed. B lymphocytes isolated from the proband showed hypersensitive Ca^2+^ responses to isoflurane and caffeine, resulting in an abnormal Ca^2+^ release only in the proband, but not in his family members. As a result, two independent ex vivo studies all show the release of Ca^2+^ in response to the RYR1 agonist (Figure 3). Ex vivo functional study using B lymphocytes supports the damaging effect of this rare *RYR1* variant classified as PS3_Supporting by modified ACMG-AMP criteria suggested for autosomal dominantly inherited RYR1/MH [23]. Therefore, the *RYR1* p.Ser2300Pro variant seems the most likely genetic cause of the clinical manifestations associated with susceptibility to MH in the proband.

## 5. Discussion

MH is a rare anesthetic emergency. It has been estimated to occur in between 1:10,000 and 1:150,000 general anesthetics [24]. One reason that diagnosis may be delayed is if the anesthetist incorrectly assumes that a history of uneventful anesthesia precludes the possibility that the patient is at risk of developing MH [25]. The potential for any of the potent inhalational anesthetics to trigger an MH reaction. Indeed, the number of cases triggered by each of the inhalational anesthetics reflects the overall usage of that particular agent [5]. Furthermore, the patient and their family should be informed about the suspected diagnosis of MH before discharge from the hospital. They should be specifically advised to warn all blood relatives of the patient that can be contacted about the risk of MH and the need to mention this should any member of the family require admission to hospital. Each member of the family should be advised that this information applies to them until it is proved otherwise using definitive diagnostic tests [25].

This study showed some differences in the genetic and pathological aspects compared to previous studies. RyR1 can mediate the release of Ca^2+^ from intracellular pools in response to nerve stimulation. It plays a crucial role in excitation-contraction coupling [26]. Classically in the *RYR1* sequence, three hot spots are considered: MH1 and MH2 domains as the large hydrophilic domain and MH3 as the C-terminal hydrophobic domain. Mutations in the recessive central core and multi-minicore myopathies are more extensively distributed along the *RYR1* sequence, whereas most heterozygous dominant mutations in central core myopathy are mapped to the C-terminal domain [27]. However, in this study, the novel pSer2300Pro variant substituted a conserved serine residue that mapped to the MH2 domain of the protein, a hot spot for MH mutations [28,29], despite being a hetero-dominant mutation. Ex vivo functional study using B lymphocytes supporting the deleterious effect of this rare *RYR1* variant contributes to an appropriate classification of variant pathogenicity as PS3_Supporting by modified ACMG-AMP criteria from PM2 by original ACMG-AMP criteria. Furthermore, although MH has previously been associated with multi-minicore disease, the cases identified as multi-minicore myopathy were exclusively linked to recessive mutations [30,31,32,33] and recently, in patients with fiber-type disproportion as their only pathological feature [34]. Interestingly, multi-minicore myopathy was observed in muscle biopsies by SDH histochemical staining in our dominant RYR1-related case. It is possible that this pathologic finding may change to the central core in adults because a previous report showed pathological findings that changed from multi-minicore to central core according to age in *RYR*-related myopathy [31].

Currently, there are two diagnostic approaches for patients potentially at increased risk of developing an MH reaction. These include molecular genetic testing and ex vivo muscle contracture testing, the in vitro contracture test (IVCT) [35] and the caffeine-halothane contracture test (CHCT) [36]. The contracture test measures the contracture response of excised skeletal muscle strips to caffeine and halothane and is regarded as the “gold standard” diagnostic test for MH susceptibility. The IVCT has a sensitivity of 100% and a specificity of 94%, whereas the CHCT has a sensitivity of 97% and a specificity of 78% [37]. The disadvantages of contracture testing are the need for specialized testing centers with trained personnel, its invasiveness, and its high cost [38]. The ryanodine receptor on B lymphocytes is identical to RyR1, which controls the calcium channel in skeletal muscle. When exposed to 4-chloro-m-cresol (4CmC), B lymphocyte ryanodine receptors can cause an acute increase in intracellular calcium [39]. Significantly higher intracellular calcium in B lymphocytes in MH-susceptible and MH-normal examinees was reported when caffeine and 4CmC were introduced [40]. In our study, Ca^2+^ responses to isoflurane and caffeine in B lymphocytes showed significant differences between the proband carrying the *RYR1* p.Ser2300Pro variant (MH-susceptible) and his family members (MH-normal). Furthermore, molecular diagnosis revealed the deleterious effect of this rare *RYR1* variant classified as PS3_Supporting by modified ACMG-AMP criteria. These results suggest that enhanced Ca^2+^ responses are associated with mutations in the *RYR1* gene in MH-susceptible individuals. Although the criteria for MH diagnosis have not been established and further comparative studies are required, RYR1-mediated intracellular Ca^2+^ release testing in B lymphocytes might have promise as an adjunct to in vitro muscle biopsies.

On the other hand, *RYR1* and *CACNA1S* as two major MH-causative genes, are huge, containing numerous exons. Mutations in these two genes account for 50–70% of the known MH cases. A comprehensive analysis by traditional Sanger sequencing is challenging because it is costly, time-consuming, and labor-intensive, without even considering other possible genes in the genome [7,9,10]. Next-generation sequencing (NGS) analysis can help delineate the genetic diagnosis of MH and several MH-susceptible clinical presentations [41,42]. Moreover, the use of NGS in unselected cohorts is an important tool to understand the prevalence and penetrance of MH susceptibility, a critical challenge in the field [43,44].

## 6. Conclusions

In conclusion, we reported a novel heterozygous *RYR1* p.Ser2300Pro variant classified as PS3_Supporting by modified ACMG-AMP criteria, leading to MH accompanied by multi-minicore myopathy. Our findings expand the clinical and pathological spectra of information associated with MH with multi-minicore myopathy. Ex vivo functional studies such as RYR1-mediated intracellular Ca^2+^ release testing in B lymphocytes may support the damaging effect of the rare *RYR1* variant.

## Figures and Tables

**Table 1:** Heterozygous missense variants identified as a genetic cause of hereditary myopathy in the proband.

Gene	Genomic Position	Ref ID	Base Change /Codon Change	rsID	OMIM	gnomAD	Inheritance
*RYR1*	chr19:38989754	NM_000540.2	c.6898T > C /p.Ser2300Pro	None	#145600, #117000	0	Sporadic
GAA *	chr17:78081415	NM_000152.5	c.752C > T /p. Ser251Leu	rs200856561	#232300	0.0003504	Maternal
GAA *	chr17:78081424	NM_000152.5	c.761C > T /p. Ser254Leu	rs577915581	#232300	0.0002071	Maternal
*GNE*	chr9:36246117	NM_001128227.3	c.620A > T /p. Asp207Val	rs139425890	#605820	0.00005173	Paternal

Human genome reference build used for genomic position: GRCh38/hg38. * The variants are found in *cis*: c.[752C > T;761C > T] p.[Ser251Leu;Ser254Leu]. Ref ID, Reference transcript ID; rsID, Reference SNP cluster ID; OMIM, Online Mendelian Inheritance in Man; #, a number sign in OMIM; gnomAD, The Genome Aggregation Database v.2.1.1 exomes.

## Data Availability

Not applicable.

## Supplementary Materials

The following are available online at https://www.mdpi.com/article/10.3390/genes13101726/s1, Table S1: A list of 293 selected genes in a Celemics G-Mendeliome Hereditary Myopathy Panel.
