# Supplementary material for: Gene Panel Sequencing Identifies a Novel RYR1 p.Ser2300Pro Variant as Candidate for Malignant Hyperthermia with Multi-Minicore Myopathy
Source: Genes (Basel). 2022 Sep 26;13(10):1726. doi: 10.3390/genes13101726 (PMC9601532; doi:10.3390/genes13101726)
Supplement: Supplementary file 1 [file genes-13-01726-s001.zip › genes-1895524-supplementary.pdf]

Supplementary Table S1. A list of 293 selected genes in a Celemics G-Mendeliome Hereditary Myopathy Panel

AARS	*ABCB7*	*ABCD1*	*ABHD12*	*ACAD9*	*ACADL*	*ACADM*	*ACO2*	*ACTA1*
ADCK3	*AFG3L2*	*AGL*	*AIFM1*	*ALDH3A2*	*AMPD1*	*ANO10*	*ANO5*	*AP4B1*
AP4E1	*AP4M1*	*AP4S1*	*AP5Z1*	*APTX*	*ARSA*	*ATCAY*	*ATL1*	*ATM*
ATP2A1	*ATP7A*	*ATP7B*	*ATP8A2*	*BAG3*	*BEAN1*	*BIN1*	*BSCL2*	*C10orf2*
C12orf65	*C19orf12*	*CACNA1A*	*CACNA1S*	*CACNB4*	*CAPN3*	*CASK*	*CAV3*	*CCDC78*
CCDC88C	*CFL2*	*CHAT*	*CHRNA1*	*CHRNB1*	*CHRND*	*CHRNE*	*CHRNG*	*CLCN1*
CLCN2	*CLN5*	*CNTN1*	*COL6A1*	*COL6A2*	*COL6A3*	*COLQ*	*CPT1B*	*CPT2*
CRYAB	*CTDP1*	*CWF19L1*	*CYP27A1*	*CYP2U1*	*CYP7B1*	*DAG1*	*DCTN1*	*DDHD1*
DDHD2	*DES*	*DMD*	*DNAJB2*	*DNAJB6*	*DNM2*	*DNMT1*	*DOK7*	*DYNC1H1*
DYSF	*EEF2*	*EGR2*	*ELOVL4*	*ELOVL5*	*EMD*	*ERLIN2*	*ETFA*	*ETFB*
FA2H	*FAM134B*	*FGD4*	*FGF14*	*FHL1*	*FIG4*	*FKRP*	*FKTN*	*FLNC*
FLVCR1	*FRMD7*	*FUS*	*FXN*	*GAA*	*GAD1*	*GALC*	*GAN*	*GARS*
GBA2	*GDAP1*	*GJB1*	*GJC2*	*GLA*	*GLE1*	*GNB4*	*GNE*	*GOSR2*
GPR143	*GRID2*	*GRM1*	*GYS1*	*HADHA*	*HADHB*	*HINT1*	*HOXD10*	*HSPB1*
HSPB8	*HSPD1*	*HSPG2*	*IGHMBP2*	*IKBKAP*	*ISPD*	*ITGA7*	*ITPR1*	*JPH3*
KBTBD13	*KCNA1*	*KCNC3*	*KCND3*	*KCNE3*	*KCNJ10*	*KCNJ18*	*KIAA0196*	*KIF1A*
KIF1B	*KIF1C*	*KIF5A*	*KLHL40*	*KLHL41*	*L1CAM*	*LAMA1*	*LAMA2*	*LARGE*
LDB3	*LITAF*	*LMNA*	*LPIN1*	*LRSAM1*	*MARS*	*MARS2*	*MATR3*	*MED25*
MFN2	*MPZ*	*MRE11A*	*MTM1*	*MTMR14*	*MTMR2*	*MTPAP*	*MTTP*	*MUSK*
MYF6	*MYH2*	*MYH7*	*MYOT*	*NDRG1*	*NEB*	*NEFL*	*NGF*	*NIPA1*
NOP56	*NTRK1*	*OPA1*	*OPA3*	*OPHN1*	*PABPN1*	*PANK2*	*PDK3*	*PDYN*
PEX7	*PFKM*	*PGAM2*	*PHKA1*	*PHYH*	*PLEC*	*PLEKHG5*	*PLP1*	*PMM2*
PMP22	*PNKP*	*PNPLA6*	*POLG*	*POLG2*	*POMGNT1*	*POMT1*	*POMT2*	*PRKCG*
PRPS1	*PRX*	*PTF1A*	*PTRF*	*PYGM*	*RAB7A*	*RAPSN*	*REEP1*	*RNF216*
RRM2B	*RTN2*	*RUBCN*	*RYR1*	*RYR2*	*SACS*	*SBF2*	*SCN4A*	*SCN9A*
SEPN1	*SETX*	*SGCA*	*SGCB*	*SGCD*	*SGCE*	*SGCG*	*SH3TC2*	*SIL1*
SLC12A6	*SLC16A2*	*SLC1A3*	*SLC33A1*	*SLC39A4*	*SLC52A2*	*SLC9A1*	*SLC9A6*	*SMN1*
SNX14	*SOD1*	*SPAST*	*SPG11*	*SPG20*	*SPG21*	*SPG7*	*SPTBN2*	*SPTLC1*
SPTLC2	*STAC3*	*STUB1*	*SUCLA2*	*SYNE1*	*SYNE2*	*SYT14*	*TBP*	*TCAP*
TDP1	*TECPR2*	*TGM6*	*TK2*	*TMEM240*	*TNNI2*	*TNNT1*	*TPM2*	*TPM3*
TPP1	*TRIM32*	*TRPV4*	*TTBK2*	*TTN*	*TTPA*	*TTR*	*TUBB4A*	*TYMP*
VAMP1	*VCP*	*VLDLR*	*VPS13A*	*VPS37A*	*VRK1*	*WFS1*	*WNK1*	*WWOX*
XK	*YARS*	*ZFYVE26*	*ZFYVE27*	*ZNF592*				
